# Antibiofilm and Antivirulence Efficacies of Flavonoids and Curcumin Against *Acinetobacter baumannii*

**DOI:** 10.3389/fmicb.2019.00990

**Published:** 2019-05-08

**Authors:** Chaitany Jayprakash Raorane, Jin-Hyung Lee, Yong-Guy Kim, Satish Kumar Rajasekharan, Rodolfo García-Contreras, Jintae Lee

**Affiliations:** ^1^School of Chemical Engineering, Yeungnam University, Gyeongsan, South Korea; ^2^Department of Microbiology and Parasitology, Faculty of Medicine, National Autonomous University of Mexico, Mexico City, Mexico

**Keywords:** *Acinetobacter baumannii*, biofilm formation, curcumin, flavonoids, mixed biofilms, motility

## Abstract

*Acinetobacter baumannii* is well adapted to hospital environments, and the persistence of its chronic infections is mainly due to its ability to form biofilms resistant to conventional antibiotics and host immune systems. Hence, the inhibitions of biofilm formation and virulence characteristics provide other means of addressing infections. In this study, the antibiofilm activities of twelve flavonoids were initially investigated. Three most active flavonoids, namely, fisetin, phloretin, and curcumin, dose-dependently inhibited biofilm formation by a reference *A. baumannii* strain and by several clinical isolates, including four multidrug-resistant isolates. Furthermore, the antibiofilm activity of curcumin (the most active flavonoid) was greater than that of the well-known biofilm inhibitor gallium nitrate. Curcumin inhibited pellicle formation and the surface motility of *A. baumannii*. Interestingly, curcumin also showed antibiofilm activity against *Candida albicans* and mixed cultures of *C. albicans* and *A. baumannii*. *In silico* molecular docking of the biofilm response regulator BfmR showed that the binding efficacy of flavonoids with BfmR was correlated with antibiofilm efficacy. In addition, curcumin treatment diminished *A. baumannii* virulence in an *in vivo Caenorhabditis elegans* model without cytotoxicity. The study shows curcumin and other flavonoids have potential for controlling biofilm formation by and the virulence of *A. baumannii*.

## Introduction

Most bacteria are able to form biofilms on various biotic and abiotic surfaces, and these films constitute structurally complex systems that defend microbial communities. Biofilm formation is a common cause of persistent infections by bacteria (Costerton et al., 1999), and resistance to eradication and high tolerance of conventional antimicrobial treatments are characteristic of bacterial biofilms (Wu et al., 2015; Lee et al., 2018).

*Acinetobacter baumannii* has been documented to be the most successful indigenous pathogen in healthcare institutions (Howard et al., 2012; Pakharukova et al., 2018). *A. baumannii* is an opportunistic Gram-negative bacillus that is responsible for a variety of nosocomial infections with high morbidity and mortality rates, these include, pneumonia, wound infections, bloodstream infections, urinary tract infections, and secondary meningitis (Howard et al., 2012; Liu et al., 2016). Furthermore, in intensive care neonatal and burns units, *A. baumannii* is one of the most commonly encountered pathogens (Seifert et al., 1994) (a claim shared with *Pseudomonas aeruginosa* and *Staphylococcus aureus*) (Paling et al., 2017a, b). Drug-resistant biofilm formation appears to play a vital role in the pathogenicity of *A*. *baumannii* (Qi et al., 2016), and biofilm development is critically dependent on the assembly of the *csuA/BABCDE* chaperon–usher, whereas pili production is required for adhesion to abiotic surfaces (Pakharukova et al., 2018). Furthermore, in *A*. *baumannii* it has been reported that biofilm formation and pili production were abolished by inactivation of the *csuE* gene (Tomaras et al., 2003), and that biofilm formation and motility are under the direct control of the two-component response regulator BfmR, which acts as a master control switch for biofilm development (Russo et al., 2016).

Flavonoids are omnipresent in the plant kingdom and exhibit antioxidative, anti-inflammatory, anti-mutagenic, and anti-carcinogenic effects (Panche et al., 2016), that coupled with metal chelation and scavenge of free radicals (Abuelsaad et al., 2014). Recently, curcumin and several other flavonoids were reported to inhibit biofilm formation by *Streptococcus mutans* (Duarte et al., 2006), *Aeromonas hydrophila* (Abuelsaad et al., 2014), *Candida albicans* (Alalwan et al., 2017), *S*. *aureus* (Lee et al., 2012), and *Escherichia coli* O157:H7 (Lee et al., 2011) and persister cells formation in *A. baumannii* (Kaur et al., 2018). However, the antibiofilm activities of flavonoids have not been investigated against *A*. *baumannii*.

In this study, twelve flavonoids initially screened for nontoxic biofilm inhibitors against *A*. *baumannii* ATCC 17978, and the effects of three active biofilm inhibitors were further investigated with eight *A*. *baumannii* clinical isolates. In order to investigate the antibiofilm efficacy of the most active curcumin, confocal laser scanning microscopy (CLSM) and scanning electron microscopy (SEM) were utilized. Also, the effect of curcumin on pellicle formation and motility was studied. In addition, antibiofilm activity of curcumin was studied in two dual species biofilm models of *C. albicans* and *A. baumannii*. Furthermore, an *in vivo Caenorhabditis elegans* model was used to study the effect of curcumin on *A*. *baumannii* virulence.

## Materials and Methods

### Ethics Statement

This study does not involve any human or animal participants nor does the study involve any invasion of privacy or accessing confidential information of individuals. The ethical committee of Yeungnam University has granted the exemption of ethical approval.

### Bacterial Strain and Chemicals

**FIGURE 1:**
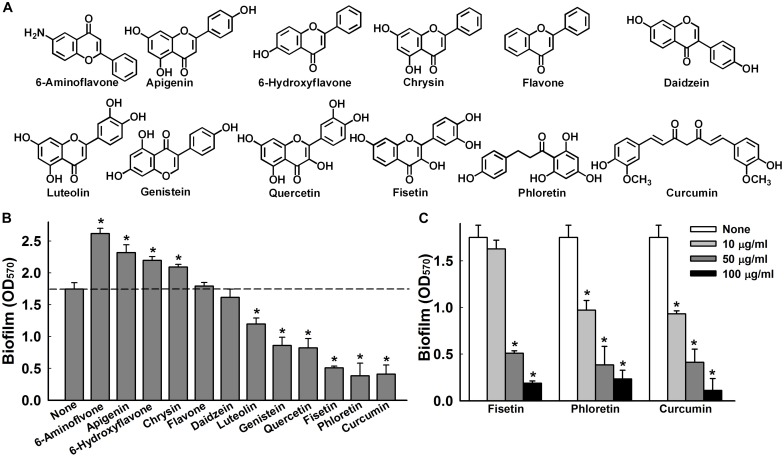
Effects of flavonoids on *A*. *baumannii* biofilm formation. Chemical structures of the flavonoids used in this study **(A)**. Effect of flavonoids on *A. baumannii* ATCC 17978 biofilm formation in TSB medium at 37°C after 24 h in 96-well plates. Total biofilm formation (OD_570_) in the presence of each flavonoid. All flavonoids were used at 50 μg/ml, except luteolin, which was used at 25 μg/ml because of its antimicrobial activity **(B)**. Dose-dependent effects of fisetin, phloretin, and curcumin on *A. baumannii* ATCC 17978 biofilm formation **(C)**. *p < 0.05 versus untreated controls.

*A. baumannii* ATCC 17978 and eight clinical *A*. *baumannii* isolates (ATCC BAA-1709, A 550, A 578, A 553, A 556, A 580, A 571, A 564) were obtained from burns patients at the National Rehabilitation Institute of Mexico; *A*. *baumannii* ATCC 17978 was used as a reference strain (Cruz-Muniz et al., 2017). For the dual biofilm experiment, we used *C*. *albicans* DAY185 (obtained from the Korean Culture Center of Microorganisms^1^) and *A*. *baumannii* ATCC 17978. All experiments were conducted at 37°C, and trypticase soy broth (TSB) and potato dextrose broth (PDB) media were used for the biofilm assay, Luria-Bertani (LB) medium for the pellicle assay, and motility agar (MA) medium in the motility experiment. Chemicals including twelve flavonoids viz. flavone (99%), 6-aminoflavone (97%), 6-hydroxyflavone (98%), apigenin (97%), chrysin (97%), curcumin (94%), daidzein (98%), fisetin (98%), genistein (98%), luteolin (98%), phloretin (99%), and quercetin (98%), gallium nitrate (99.9%), and crystal violet (90%) were purchased from Sigma-Aldrich Co. (MO, United States). The structures of these flavonoids are provided in Figure 1A. TSB, PDB, LB media, and ethanol (95%) were purchased from Becton Dickison and company (NJ, United States) and dimethyl sulfoxide (DMSO) from Duksan Pure Chemicals (Daegu, South Korea), respectively. All 12 flavonoids solutions were prepared by diluting them in DMSO that was also used as a negative control.

### Bacterial Culture and Minimum Inhibitory Concentration (MIC) Assay

*A. baumannii* initially was streaked from −80°C glycerol stock on trypticase soy agar (TSA) plates, and a single fresh colony was inoculated in TSB (2 ml) in 14-ml tubes and incubated at 37°C and 250 rpm (Lee et al., 2012). Overnight cultures were reinoculated at 1:100 dilution in TSB. For cell growth measurements, a 1:100 inoculum was cultured in TSB (25 ml) in 250 ml flasks and incubated at 37°C overnight with agitation at 250 rpm. Optical densities were then measured at 600 nm using a spectrophotometer (Optizen 2120 UV, Mecasys, South Korea). Streaking and subculturing of *C*. *albicans* DAY185 was performed using potato dextrose agar (PDA) or PDB, unless otherwise specified (Manoharan et al., 2017). *C. albicans* was preserved at −80°C in 1 ml of PDB supplemented with 30% glycerol, and when needed, streaked on PDA plates. Plates were incubated for 48 h at 37°C and a fresh single colony was then inoculated into 25 ml of PDB and cultured overnight at 37°C. A minimum two independent cultures were used for each experiment.

MICs were determined (Betts and Wareham, 2014; Singh, 2014; Singh et al., 2017) using the National Committee for Clinical Laboratory Standards (2002) susceptibility testing guidelines in 96-well microtiter plates (SPL Life Sciences, Pocheon, South Korea). Briefly, an overnight culture at a dilution of 1:100 inoculated in TSB was cultured for 24 h in the presence of curcumin (100, 200, or 500 μg/ml) at 37°C. After incubation cultures were spread on TSA plates, incubated for 24 h at 37°C, and cell colonies were counted. Experiments were performed using at least two independent cultures.

### Crystal Violet Biofilm Assay and Antibiofilm Screening

Static biofilm formation was assayed in 96-well polystyrene plates as previously reported (Lee et al., 2014). Briefly, cells in TSB (total volume 300 μl) inoculated at initial turbidity of 0.05 at 600 nm (OD_600_) were cultured with or without for 24 h without shaking at 37°C. The same amount of TSB was added to peripheral wells of 96-well plate to avoid edge effects. To quantify total biofilm formation, biofilms in 96-well plates were stained with 0.1% crystal violet for 20 min, dissolved in 95% ethanol, and absorbances were measured at 570 nm (OD_570_). Cell growth in 96-well plates was also measured at 620 nm (OD_620_). For initial anti-biofilm screening, we tested all twelve flavonoids at a concentration of 50 μg/ml; results presented are averages of at least six replicate wells. Another static biofilm formation assay was performed in 14 ml polyethylene tube, as previously described (Pour et al., 2011). *A. baumannii* ATCC 17978 cells were inoculated 1:100 in 5 ml of TSB medium with curcumin at 0, 10, 20, 50, and 100 μg/ml and incubated for 24 h without shaking. Ring biofilms in 14 ml polyethylene tubes were stained with crystal violet and results presented are the averages of at least three repetitions.

### Confocal Laser Scanning Microscopy

*Acinetobacter baumannii* was inoculated at an OD_600_ of 0.05 in 3 ml of TSB in glass bottomed confocal dishes (SPL life Sciences, Pocheon, South Korea) for 24 h at 37°C with curcumin at 0, 10, and 50 μg/ml without shaking. To visualize biofilm structures, cells were stained with carboxyfluorescein diacetate succinimidyl ester (Invitrogen, Molecular Probes, Inc, Eugene, OR, United States). Biofilm structures were evaluated by CLSM (Nikon Eclipse Ti, Tokyo, Japan) (Lee et al., 2014), and their spatial characteristics were quantified using COMSTAT biofilm program^2^ by analyzing at least four random positions in three independent cultures. To measure biofilm formation, color confocal images (20 image stacks) were converted to gray scale using ImageJ program^3^. COMSTAT biofilm software was used to determine biomasses (μm^3^ per μm^2^), mean thicknesses (μm), and substratum coverages (%) (Runci et al., 2017).

### Assessment of Pellicle Formation

*A. baumannii* can form pellicles more readily at air-liquid interfaces than other pathogenic *Acinetobacter* species (Chabane et al., 2014). The pellicle formation assay used was a modification of a previously described protocol (Marti et al., 2011). In brief, overnight bacterial cultures were diluted 1:100 in 5 ml of LB broth and grown in glass tubes for 72 h at 25 and 37°C in the dark without agitation (Mussi et al., 2010). Amounts of pellicle material were assessed by adding 1 ml of ethanol to tube underneath pellicle material, removing floating pellicles, and resuspending them in phosphate buffer saline (PBS) as previously reported (Giles et al., 2015). OD_600_ values were measured using a spectrophotometer (Optizen 2120 UV, Mecasys, South Korea). Experiments were conducted in triplicate on three different days.

### Surface Motility Assay

To assess surface motility with different concentrations of agar, MA containing 0.4% agarose, 1% tryptone, and 0.5% yeast extract was used (Clemmer et al., 2011), and MA medium supplemented with 0.25% agar (Eijkelkamp et al., 2011). Curcumin at 10 and 50 μg/ml concentration was added to MA, and DMSO (0.1%) was used as a negative control. Overnight grown ∼0.2 μl cultures of *A*. *baumannii* ATCC 17978 and three other multi-drug resistant clinical isolates (A 550, A 556, and A 580) were placed on motility plates using a sterile pipette tip. Sizes of halos produced by cells traveling across agar plates were measured after 9 h of incubation at 37°C. Each experiment was performed using at least three independent cultures.

### Mixed Culture Biofilm Assay

Because of its antibiofilm activity against *C*. *albicans* (Alalwan et al., 2017; Tan et al., 2018), we speculated curcumin would exhibit antibiofilm activity against a mixed culture of *C*. *albicans* and *A*. *baumannii*. Cells were inoculated together in PDB and TSB (50:50) mixed for *C. albicans* and *A. baumannii* at 1:50 (CFU ∼1 × 10^7^) and 1:100 (CFU ∼7 × 10^7^) dilution ratios from each with overnight cultures, respectively. Pure cultures of *C. albicans* and *A. baumannii* were tested at the same time. Biofilms in 96-well plates were stained with 0.1% crystal violet, dissolved 95% ethanol, and OD_570_ values were used to quantify total biofilm formation. Cell growth in 96-well plate were determined using OD_620_ values. Hyphal formation by *C*. *albicans* and mixed biofilm formation were assessed by SEM as previously described (Lee et al., 2014). Briefly, small pieces (0.5 cm × 0.5 cm) of nylon filter were placed in wells of 96-well plates containing 300 μl cells/well. Cells were incubated in the absence or presence of curcumin at 37°C for 24 h without shaking. Prior to observation, biofilm samples were fixed with 2.5% glutaraldehyde and 2% formaldehyde for 24 h, serially post fixed in PBS and osmium tetroxide, and dehydrated using an ethanol series (50, 70, 80, 90, 95, and 100%) and isoamyl acetate. After critical-point drying, cells on filters were sputter-coated with palladium/gold and observed under an S-4100 scanning electron microscope (Hitachi, Tokyo, Japan) at magnifications ranging from x 1,000 to 10,000 using an accelerating voltage of 15 kV.

### Molecular Docking Simulations of Flavonoids With BfmR

The molecular docking assays was conducted as previously described (Russo et al., 2016; Rajasekharan et al., 2017). Docking studies were performed to evaluate interactions between all twelve flavonoids and BfmR binding sites of *A*. *baumannii* (Protein Data Bank 6BR7). The three-dimensional structure of the beryllium fluorinated (BeF_3_^–^) receiver domain of *A*. *baumannii* BfmR resolved at 1.86 Å was used for docking simulation. This BfmR domain consisted of two chains (A and B) and has a sequence length of 133 amino acids. For grid generation, beryllium fluorinated ligand was used as the centroid and ligands were docked at positions proximal to BeF_3_^–^ binding pockets (Draughn et al., 2018) using Schrodinger software 11.4 (Cambridge, MA, United States). A BfmR inhibitor 2-aminoimidazole and two biofilm inhibitors, virstatin and LED 209, were also docked at active sites. The glide score value and more specific target binding interactions to Asp15 and Asp58 were recorded.

### *C. elegans* Killing Assay

The *C. elegans* killing assay used was a modification of a previously described protocol (Beceiro et al., 2014). Briefly, non-infected nematodes (∼20–30) [*fer-15(b26);fem-1(hc17)*] were pipetted into 96-well plate containing M9 buffer and overnight curcumin (50 μg/ml) treated and untreated with *A. baumannii* and/or *C. albicans* cells. As a second dose curcumin was added to respective wells to make final concentration 50 μg/ml (total volume 300 μl). Nematodes were incubated at 25°C and viabilities were determined as previously described (Rajsekharan et al., 2018), by exposing them to LED or UV LED lights for 10–30 s using an iRiS^TM^ Digital Cell Imaging System (Logos BioSystems, South Korea). Three independent experiments (*n* = ∼20–30) were conducted.

### Statistical Analysis

Replication numbers for assays are provided above and results are expressed as means ± standard deviations. The statistical analysis was performed by one-way ANOVA followed by Dunnett’s test using SPSS version 23 (SPSS Inc., Chicago, IL, United States). *P* values of < 0.05 were regarded significant and asterisks are used to indicate significant differences between treated and untreated samples.

## Results

### Impacts of Flavonoids on Biofilm Formation by *A. baumannii*

The effects of the 12 flavonoids (Figure 1A) were initially investigated on *A*. *baumannii* ATCC 17978 biofilm formation in 96-well polystyrene plates using a crystal violet assay. Of the 12 flavonoids, luteolin, genistein, quercetin, fisetin, phloretin, and curcumin at 50 μg/ml exhibited biofilm inhibition, whereas 6-aminoflavone, apigenin, 6-hydroxyflavone, and chrysin increased biofilm formation, and the backbone flavone and daidzein had little effect (Figure 1B). Three flavonoids, that is, fisetin, phloretin, and curcumin, significantly and dose-dependently reduced biofilm formation, for example, these three flavonoids at 10 and 100 μg/ml reduced biofilm formation by *A*. *baumannii* ATCC 17978 by >45 and >86%, respectively (Figure 1C).

### Antibiofilm Activities of Fisetin, Phloretin, and Curcumin Against *A. baumannii* Clinical Isolates

**FIGURE 2:**
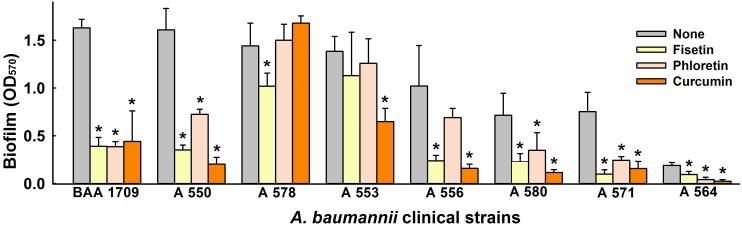
Inhibitory effect of lead flavonoids on biofilm formation by *A*. *baumannii* clinical isolates. Biofilm formations by *A*. *baumannii* clinical isolates (ATCC BAA-1709, A 550, A 578, A 553, A 556, A 580, A 571, A 564) were quantified in the presence of fisetin, phloretin, or curcumin at 50 μg/ml after 24 h in 96-well plates. *p < 0.05 versus untreated controls.

Next, we investigated the antibiofilm activities of fisetin, phloretin, and curcumin against eight clinical *A*. *baumannii* strains (Cruz-Muniz et al., 2017). Of these strains, ATCC BAA-1709, A 550, A 578, and A 553 were highly biofilm-forming, A 556, A 580, A 571 were intermediate, and A 564 had poor biofilm forming ability. Interestingly, fisetin, phloretin, and curcumin at 50 μg/ml all inhibited biofilm formation by the multidrug resistance (MDR) strains A 550, A 556, A 580, and A 564, and the antibiotic-sensitive ATCC BAA-1709 and A 571 strains. On the other hand, biofilm formation by two MDR strains, that is, A 553 (sensitive to colistin and amikacin), and A 578 (sensitive to colistin, imipenem, and meropenem) were not affected by fisetin, phloretin, or curcumin (Figure 2). Of these three flavonoids, curcumin was the most effective biofilm inhibitor against tested *A*. *baumannii* strains, and thus, it was the focus of subsequent studies conducted using the reference *A*. *baumannii* ATCC 17978 strain.

### Effects of Curcumin on Planktonic Cell Growth and Biofilm Formation

**FIGURE 3:**
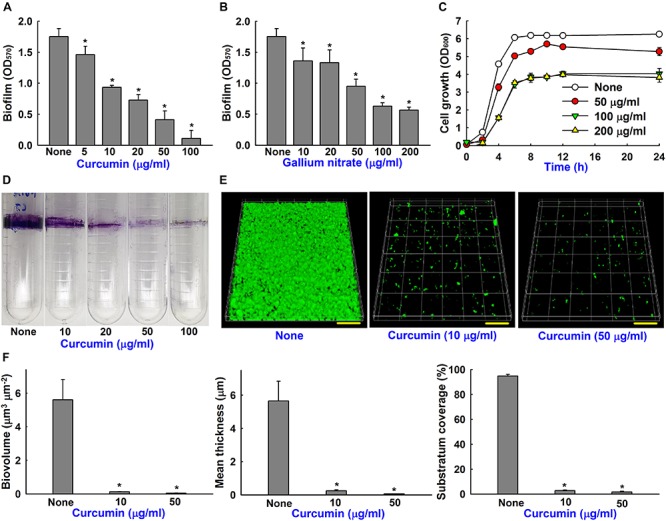
Biofilm inhibition of *A*. *baumannii* by curcumin and gallium nitrate. Dose-dependent effects of curcumin on biofilm formation by *A. baumannii* ATCC 17978 **(A)**. Effect of gallium nitrate on biofilm formation **(B)**. Effect of curcumin on the cell growth of *A. baumannii*. Planktonic cell growth of *A. baumannii* was measured at 600 nm in 250 ml flasks stirred at 250 rpm **(C)**. Dose-dependent effect of curcumin on *A. baumannii* ring biofilm formation on polyethylene when incubated at 37°C under static conditions **(D)**. CLSM observation of biofilm inhibition by curcumin **(E)**. Scale bar = 50 μm. Biofilm biomasses, mean thicknesses and substratum coverages spatial characteristics were quantified by COMSTAT analysis **(F)**. *p < 0.05 versus untreated controls.

The antibiofilm activity of curcumin was compared with that of gallium nitrate (a known biofilm inhibitor) (Runci et al., 2017). Both curcumin and gallium nitrate dose-dependently inhibited biofilm formation by the ATCC 17978 strain, though curcumin was superior to gallium nitrate at same concentrations (Figures 3A,B). For example, curcumin at 20 or 100 μg/ml decreased biofilm formation in polystyrene 96-well plate by 46 and 93%, respectively, whereas gallium nitrate at these concentrations decreased biofilm formation by 24 and 67%, respectively. Biofilm formation was also assessed in polyethylene tubes, as previously described (Pour et al., 2011), and curcumin was found to dose-dependently inhibit ring biofilm formation by *A*. *baumannii* (Figure 3D). We also examined the effect of curcumin on planktonic cell growth. The MICs of curcumin and gallium nitrate against ATCC 17978 strain were determined to be >500 and >1000 μg/ml, respectively. It was difficult to determine an exact MIC as curcumin precipitated from solution at higher concentrations, as previously reported (Betts and Wareham, 2014). Curcumin at concentrations up to 200 μg/ml slightly reduced (by ≤38%) the planktonic cell growth of *A*. *baumannii* under shaking conditions in a flask (Figure 3C). These results indicated that antibiofilm activity of curcumin was not due to its antimicrobial activity, indicating curcumin may less prone to the development of drug resistance.

### Microscopic Observations of Biofilm Inhibition by Curcumin

The antibiofilm effect of curcumin was further confirmed by CSLM and COMSTAT analysis. Interestingly, *A*. *baumannii* ATCC 17978 formed relatively thin surface biofilms on glass surfaces (Figure 3E) but robust ring biofilms on polyethylene (Figure 3D). Furthermore, the antibiofilm activity of curcumin was more marked on glass than polystyrene (Figure 3A) and polyethylene (Figure 3D) surfaces. For example, curcumin at 10 μg/ml markedly reduced surface biofilm formation on glass (Figure 3E), whereas at 100 μg/ml curcumin was more effective at preventing ring biofilm formation on polyethylene (Figure 3D). Biofilm reduction was also confirmed by COMSTAT analysis, which showed curcumin at 10 or 50 μg/ml significantly reduced biofilm biomasses, average thicknesses, and substrate coverage (Figure 3F). Specifically, biofilm biomass, thickness, and substrate coverage were reduced by curcumin at 10 μg/ml by >95% versus untreated controls.

### Inhibitory Effect of Curcumin on Pellicle Formation

**FIGURE 4:**
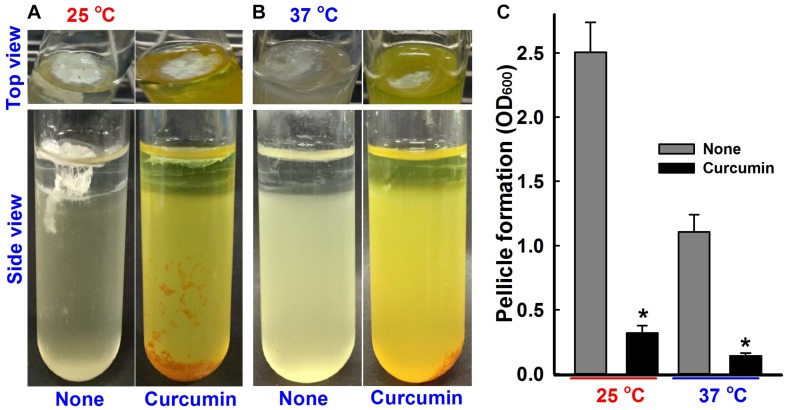
Pellicle inhibition by curcumin. *A. baumannii* ATCC 17978 strain was grown in the presence or absence of curcumin (50 μg/ml) for 72 h at 25°C **(A)** or 37°C **(B)**. Bar graphs represent pellicle formation as determined by spectrophotometry at OD_600_ **(C)**. Experiments were performed using at least two independent cultures. *p < 0.05 versus untreated controls.

*A. baumannii* colonizes the upper surfaces of static liquids and form biofilms at air-liquid interfaces by a process called pellicle formation, which is a type of biofilm formation (Kentache et al., 2017). The effect of curcumin on pellicle formation of ATCC 17978 and three other multi-drug resistant clinical isolates (A 550, A 556, and, A 580) were measured at 25 and 37°C in LB medium without shaking. After 24 h, a thin pellicle started to form at the liquid surface, and by the end of the third day, an opaque, solid pellicle covered the entire liquid surface (Figures 4A,B and Supplementary Figure S1D). Pellicle growth was found to be greater at 25°C, which concurs with the results of Marti et al. (2011), and to be significantly inhibited by curcumin at 50 μg/ml (Figure 4C and Supplementary Figure S1F).

### Inhibition of Surface Motility by Curcumin

**FIGURE 5:**
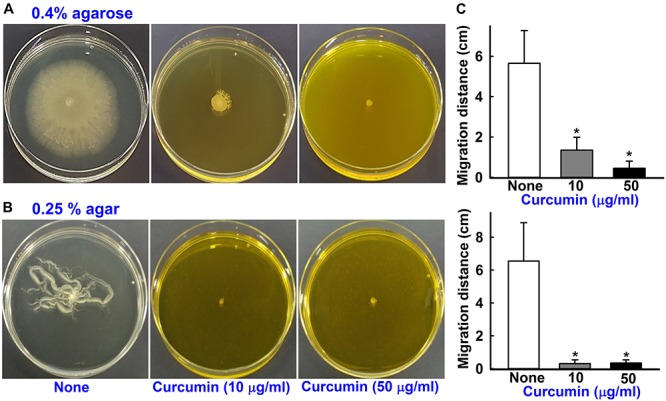
Effects of curcumin on motility. The surface motilities on 0.4% agarose **(A)** and 0.25% agar **(B)** of *A*. *baumannii* ATCC 17978 were investigated after adding curcumin at 10 or 50 μg/ml to motility agar. The bar graphs represent swimming and swarming motility diameters in cm in the presence and absence of curcumin **(C)**. *p < 0.05 versus untreated controls.

*Acinetobacter baumannii* biofilm formation depends on the synthesis of pili, which are structures assembled by the *csuA/BABCDE* chaperone-usher secretion system (Luo et al., 2015). Curcumin at 10 μg/ml reduced surface motility on 0.4% agarose and 0.25% agar (Figures 5A,B). Surface motility of ATCC 17978 with 0.4% agarose measuring mean halo diameters at 9 h were 1.4 ± 0.6 cm for curcumin treatment and 5.6 ± 1.6 cm for the non-treated control. Similarly, surface motilities measured in 0.25% agar using mean halo diameters were 0.3 ± 0.2 cm for curcumin at 10 μg/ml and 6.6 ± 2.3 cm for the control. Also, three clinical isolates (A 550, A 556, and A 580) were motile, which was significantly inhibited by curcumin (Supplementary Figures S1A–C,E). This is interesting since pili play a role both in biofilm formation and motility that were markedly abolished by curcumin.

### Mixed Culture Biofilm Inhibition by Curcumin

**FIGURE 6:**
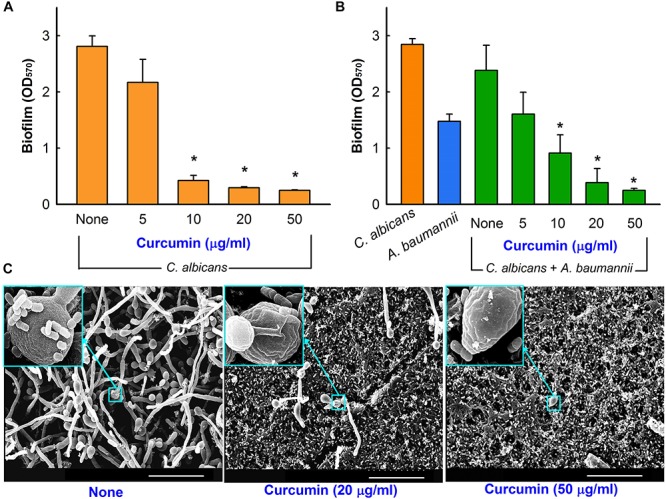
Antibiofilm activity of curcumin in the mixed biofilm model. Antibiofilm effect of curcumin against *C*. *albicans* DAY185 in PDB medium **(A)**. Antibiofilm effect of curcumin on mixed *C. albicans* DAY185 and *A. baumannii* ATCC 17978 biofilms was determined in a (50:50) mixed of PDB and TSB media after culture for 24 h in 96-well plates **(B)**. SEM observation of *C. albicans* and *A. baumannii* mixed biofilms. In insets, the larger cells are *C. albicans* and the smaller cells are *A. baumannii* **(C)**. Scale bar = 30 μm. *p < 0.05 versus untreated controls.

Biofilm formation is a survival policy for bacteria and fungi in challenging environments (Wu et al., 2015). Crystal violet biofilm and SEM assays were used to examine the inhibitory effects of curcumin on mixed biofilms of *C*. *albicans* and *A*. *baumannii*. To form these dual biofilms, we used a (50:50) mixed medium of PDB and TSB to enable *C*. *albicans* and *A*. *baumannii* growth, respectively. Under these conditions, decent biofilm formation (1.0∼3.0 at OD_570_) of individual *C. albicans* and *A*. *baumannii* strain and also co-culture of two species was observed (Figures 6A,B). As previously reported by Alalwan et al. (2017), curcumin dose-dependently inhibited biofilm formation by *C. albicans* (Figure 6A), for example, at 10 μg/ml curcumin reduced its biofilm formation by >80%. Importantly, curcumin at 20 μg/ml reduced mixed biofilm formation by >85% (Figure 6B). SEM analysis showed that in mixed biofilms of *C. albicans* and *A*. *baumannii*, *C. albicans* formed large hyphae and few yeast cells, which were much larger than *A*. *baumannii* cells, and *A*. *baumannii* cells appeared to be encased in *C. albicans* hyphae (Figure 6C).

### Interactions of Flavonoids With the Biofilm Response Regulator BfmR

**FIGURE 7:**
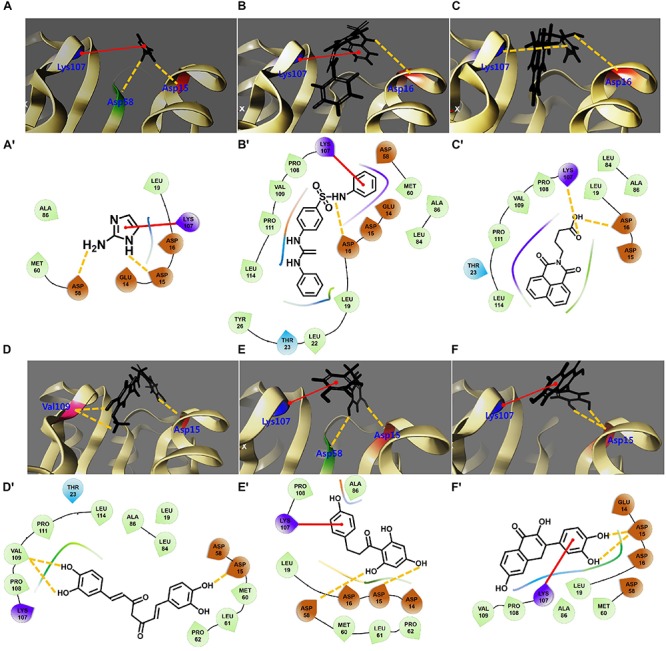
2D and 3D interaction patterns of flavonoids with the N-terminal domain of BfmR. 3D binding orientations of 2-aminoimidazole **(A)**, LED209 **(B)**, virstatin **(C)**, curcumin **(D)**, phloretin **(E)**, and fisetin **(F)** with respect to the active site of BfmR. The protein represented by ribbon. **(A′–F′)** show 2D interactions for respective ligands and surrounding amino acids residues. Negatively charged amino acids are depicted as red drops, hydrophobic amino acids as light green drops, and positively charged amino acids as violet drops. Backbone hydrogen bonds are shown as yellow dotted lines and Pi-Pi stacking is shown as red lines.

Biofilm inhibition in *A. baumannii* is under the control of a BfmR/S, which is a two-component system (Liou et al., 2014). In this part of the study, we investigated interactions between several flavonoids and the BeF_3_^–^ domain of BfmR (Figure 7 and Supplementary Figure S2). The positive control, 2-aminoimidazole (Thompson et al., 2012) exhibited hydrogen bond interactions with Asp15 and Asp58 and a Pi-Pi stacking with Lys107 (Figure 7A). Also, two known biofilm inhibitors (virstatin and LED209) interacted with Asp16 (adjacent to Asp15) and Lys107 (Figures 7B,C). Curcumin was found to interact well with the active site with a binding energy of −38.7 kcal/mol (Supplementary Table S1). It formed backbone H-bonds with negatively charged Asp15 and two H-bonds with non-polar Val109 (Figure 7D). Fistein and quercetin also formed two H-bonds with Asp15 and Pi-Pi stacking with Lys107 (Figure 7F, Supplementary Figure S2, and Supplementary Table S1), while phloretin was found to interact better than fistein or quercetin with a binding energy of −41.8 kcal/mol, resulting from the formation of H-bonds with Asp15 and Asp58 and Pi-Pi stacking with Lys107 (Figure 7E, Supplementary Figure S2, and Supplementary Table S1). Several other flavonoids (6-aminoflavone, apigenin, 6-hydroxyflavone, luteolin, chrysin, flavone, and daidzein) were also tested which did not interact with the active site and showed poor interaction patterns (Supplementary Figure S2). Furthermore, our *in vitro* studies, showed these flavonoids did not inhibit biofilm formation (Figure 1B). Overall, BfmR binding efficacies of all 12 flavonoids were correlated with their antibiofilm efficacies. Based on *in vitro* and *in silico* findings, we speculate that the interaction of curcumin with BfmR could be one of the possible causes for its antibiofilm activity. However, further *in vitro* studies are required to confirm the curcumin/BfmR interaction.

### Curcumin Increased the Survival of *C. elegans* Exposed to *A. baumannii*

**FIGURE 8:**
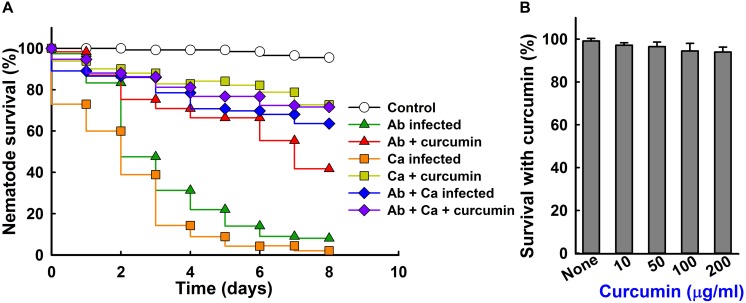
Effect of curcumin on the survival of nematodes with *A*. *baumannii* and/or *C. albicans* infection. Liquid killing assay of *C. elegans* strain *fer-15(b26);fem-1(hc17)* infected with *A*. *baumannii* ATCC 17978 and/or *C. albicans* DAY185 in the presence of curcumin **(A)**. Ab indicates *A*. *baumannii* ATCC 17978 and Ca indicates *C. albicans* DAY185. The effects of curcumin on non-infected nematodes after 4 days of exposure **(B)**.

Since *A*. *baumannii* and *C. albicans* kills the nematode *C. elegans* (Beceiro et al., 2014), a *C. elegans* killing assay was performed to examine the protective effect of curcumin. *A*. *baumannii* infection caused 80% *C. elegans* fatality (20% survival) in 5 days (Figure 8A), but the presence of curcumin at 50 μg/ml reduced this to 35% (Figure 8A and Supplementary Figure S3), while the cell numbers of *A*. *baumannii* are similar (Supplementary Figure S4). Also, curcumin significantly attenuated the virulence of *C. albicans* in the nematode. These results show that curcumin effectively reduced the virulence of *A*. *baumannii* and *C. albicans* in our nematode model. Interestingly, mixed infection of *C. albicans* and *A. baumannii* showed much less virulence than the single pathogenic infection on *C. elegans*. This result confirms that *C. albicans* and *A. baumannii* are antagonistic each other and reduce their virulence against *C. elegans* as reported in references (Kostoulias et al., 2016). In addition, we investigated the chemical toxicity of curcumin against uninfected *C*. *elegans*. After 4 days trial, curcumin treated nematodes showed similar trends like the non-treated controls (Figure 8B), confirming that curcumin was nontoxic to worms, and did not affect the survival rate.

## Discussion

Flavonoids are a ubiquitous class of phytocompounds and considered prospective candidates for drug design (Merken and Beecher, 2000). Here, we report the biofilm inhibitory potentials of several flavonoids against the clinically relevant biofilm-forming bacterial pathogen *A. baumannii*. Of the twelve flavonoids tested, curcumin, fisetin, and phloretin most efficiently reduced biofilm formation by *A. baumannii* strains, including six clinical isolates (Figures 1, 2). In particular, curcumin inhibited ring biofilm formation, pellicle formation, motility, and mixed *C. albicans* and *A. baumannii* biofilm formation (Figures 3–6). Molecular docking analysis indicated flavonoids can interact with the biofilm response regulator BfmR (Figure 7 and Supplementary Figure S2).

Curcumin is an unstable, reactive, nonbioavailable compound (Nelson et al., 2017) while it is an established therapeutic agent and is effective against various strains of Gram- negative and Gram-positive pathogens (Jaiswal and Mishra, 2018). Its mode of action at the molecular level has not been established, but it is thought to disrupt bacterial membranes (Tyagi et al., 2015; Teow et al., 2016). In the present study, we observed that at low concentrations, curcumin possibly inhibits biofilm formation by blocking BfmR, which is an interesting prospective therapeutic target in *A. baumannii*, as it has been shown inactivation of BfmR inhibits biofilm, motility and pellicle formation by *A. baumannii* (Russo et al., 2016). Recently, BfmR in *A. baumannii* was identified as a drug target, and as a result, several high-throughput molecular docking approaches have been used to identify small molecule BfmR inhibitors that interact strongly with biologically relevant sites in BfmR (Draughn et al., 2018). Similarly, we performed molecular docking to predict the binding efficacies of flavonoids with modeled BfmR. Active sites in BfmR were putatively identified by Draughn et al. (2018) who found several negatively charged amino acids in its active site. These included the conserved Asp58 and Asp15 residues coordinated with the BeF_3_^–^ domain and a Mg^2+^ ion. In the present study, we found that our lead compounds (genestein, quercetin, fisetin, phloretin, and curcumin) and the standard inhibitors (virstatin, LED209, and 2-aminoimidazole) have strong positive interactions with BfmR. However, no such interactions were observed for seven other flavonoids (luteolin, apigenin, daidzein, chrysin, flavone, 6-hydroxyflavone, and 6-aminoflavone), and these flavonoids did not exhibit antibiofilm activity (Figure 1B). Thus, our results suggest the inhibitory activities of flavonoids are related to BfmR binding modes.

In mixed culture of *A. baumannii* and *C. albicans* biofilms, the outer membrane protein of *A*. *baumannii* FhaB binds to Hyr1p (encoded by *HYR1*) of *C*. *albicans*, and it has been reported that *HYR1* knockdown significantly reduces *A*. *baumannii* binding to *C*. *albicans* hyphae (Darwish Alipour Astaneh et al., 2017; Uppuluri et al., 2018). We observed mixed cultures *C. albicans* and *A. baumannii* formed substantial amounts of biofilm and that this was dose-dependently reduced by curcumin. We hope our findings will aid the fight against polymicrobial infections and believe they have significant disease management implications because they impact antimicrobial therapy selection against drug-resistant microorganisms. Furthermore, curcumin effectively reduced *A*. *baumannii* virulence *in vivo* in our *C*. *elegans* model without exhibiting toxicity (Figure 8). These findings show curcumin is a potential candidate for antivirulence strategies against persistent *A*. *baumannii* infections.

## Conclusion

The expansion in drug resistance to conventional antibiotics has necessitated the developments of alternative antibiotic and antifungal agents. Over past decades, curcumin has been demonstrated to have potent antibiofilm activity and other pharmacological actions. Curcumin is marketed as a health supplement mainly for its antibacterial, antioxidant and anti-inflammatory properties. However, the present study, curcumin was found to inhibit biofilm formation by *A*. *baumannii* strains and by *A*. *baumannii* and *C*. *albicans* mixtures and to attenuate *A*. *baumannii* virulence in our nematode model. These findings indicate curcumin has potential use as an alternative antibiotic or antifungal agent. However, we recommend more investigations be conducted to better understand the broad action of curcumin before efforts are made to develop antibiofilm or antivirulence agents based on curcumin.

## Author Contributions

CR, J-HL, and Y-GK performed *in vitro* experiments, and analyzed the data. SR performed the docking studies. RG-C provided bacterial isolates and helped to design study. CR, J-HL, SR, and JL designed the study and wrote the manuscript. All the authors read and approved the final manuscript.

## Conflict of Interest Statement

The authors declare that the research was conducted in the absence of any commercial or financial relationships that could be construed as a potential conflict of interest.

## Footnotes

1 http://www.kccm.or.kr/

2 http://www.comstat.dk/

3 https://imagej.nih.gov/ij/

## References

[B1] Abuelsaad A. S. Allam G. Al-Solumani A. A. (2014). Hesperidin inhibits inflammatory response induced by *Aeromonas hydrophila* infection and alters CD4${}^{+}$/CD8${}^{+}$ T cell ratio. *Med. Inflamm.* 2014:393217. 10.1155/2014/393217 24891765 PMC4033591

[B2] Alalwan H. Rajendran R. Lappin D. F. Combet E. Shahzad M. Robertson D. (2017). The anti-adhesive effect of curcumin on *Candida albicans* biofilms on denture materials. *Front. Microbiol.* 8:659. 10.3389/fmicb.2017.00659 28473808 PMC5397414

[B3] Beceiro A. Moreno A. Fernandez N. Vallejo J. A. Aranda J. Adler B. (2014). Biological cost of different mechanisms of colistin resistance and their impact on virulence in *Acinetobacter baumannii*. *Antimicrob. Agents Chemother.* 58 518–526. 10.1128/AAC.01597-13 24189257 PMC3910726

[B4] Betts J. W. Wareham D. W. (2014). *In vitro* activity of curcumin in combination with epigallocatechin gallate (EGCG) versus multidrug-resistant *Acinetobacter baumannii*. *BMC Microbiol.* 14:172. 10.1186/1471-2180-14-172 24969489 PMC4083870

[B5] Chabane Y. N. Marti S. Rihouey C. Alexandre S. Hardouin J. Lesouhaitier O. (2014). Characterisation of pellicles formed by *Acinetobacter baumannii* at the air-liquid interface. *PLoS One* 9:e111660. 10.1371/journal.pone.0111660 25360550 PMC4216135

[B6] Clemmer K. M. Bonomo R. A. Rather P. N. (2011). Genetic analysis of surface motility in *Acinetobacter baumannii*. *Microbiology* 157 2534–2544. 10.1099/mic.0.049791-0 21700662 PMC3352170

[B7] Costerton J. W. Stewart P. S. Greenberg E. P. (1999). Bacterial biofilms: a common cause of persistent infections. *Science* 284 1318–1322. 10.1126/science.284.5418.1318 10334980

[B8] Cruz-Muniz M. Y. Lopez-Jacome L. E. Hernandez-Duran M. Franco-Cendejas R. Licona-Limon P. Ramos-Balderas J. L. (2017). Repurposing the anticancer drug mitomycin C for the treatment of persistent *Acinetobacter baumannii* infections. *Int. J. Antimicrob. Agents* 49 88–92. 10.1016/j.ijantimicag.2016.08.022 27939675

[B9] Darwish Alipour Astaneh S. Rasooli I. Mousavi Gargari S. L. (2017). Filamentous hemagglutinin adhesin FhaB limits *A. baumannii* biofilm formation. *Front. Biosci.* 9 266–275. 10.2741/e801 28199190

[B10] Draughn G. L. Milton M. E. Feldmann E. A. Bobay B. G. Roth B. M. Olson A. L. (2018). The structure of the biofilm-controlling response regulator BfmR from *Acinetobacter baumannii* reveals details of its DNA-binding mechanism. *J. Mol. Biol.* 430 806–821. 10.1016/j.jmb.2018.02.002 29438671 PMC5861000

[B11] Duarte S. Gregoire S. Singh A. P. Vorsa N. Schaich K. Bowen W. H. (2006). Inhibitory effects of cranberry polyphenols on formation and acidogenicity of *Streptococcus mutans* biofilms. *FEMS Microbiol. Lett.* 257 50–56. 10.1111/j.1574-6968.2006.00147.x 16553831

[B12] Eijkelkamp B. A. Stroeher U. H. Hassan K. A. Papadimitrious M. S. Paulsen I. T. Brown M. H. (2011). Adherence and motility characteristics of clinical *Acinetobacter baumannii* isolates. *FEMS Microbiol. Lett.* 323 44–51. 10.1111/j.1574-6968.2011.02362.x 22092679

[B13] Giles S. K. Stroeher U. H. Eijkelkamp B. A. Brown M. H. (2015). Identification of genes essential for pellicle formation in *Acinetobacter baumannii*. *BMC Microbiol.* 15:116. 10.1186/s12866-015-0440-6 26047954 PMC4457973

[B14] Howard A. O’donoghue M. Feeney A. Sleator R. D. (2012). *Acinetobacter baumannii*: an emerging opportunistic pathogen. *Virulence* 3 243–250. 10.4161/viru.19700 22546906 PMC3442836

[B15] Jaiswal S. Mishra P. (2018). Antimicrobial and antibiofilm activity of curcumin-silver nanoparticles with improved stability and selective toxicity to bacteria over mammalian cells. *Med. Microbiol. Immunol.* 207 39–53. 10.1007/s00430-017-0525-y 29081001

[B16] Kaur A. Sharma P. Capalash N. (2018). Curcumin alleviates persistence of *Acinetobacter baumannii* against colistin. *Sci. Rep.* 8:11029. 10.1038/s41598-018-29291-z 30038318 PMC6056455

[B17] Kentache T. Ben Abdelkrim A. Jouenne T. Dé E. Hardouin J. (2017). Global dynamic proteome study of a pellicle-forming *Acinetobacter baumannii* strain. *Mol. Cell Proteo.* 16 100–112. 10.1074/mcp.M116.061044 27799293 PMC5217776

[B18] Kostoulias X. Murray G. L. Cerqueira G. M. Kong J. B. Bantun F. Mylonakis E. (2016). Impact of a cross-kingdom signaling molecule of *Candida albicans* on *Acinetobacter baumannii* physiology. *Antimicrob. Agents Chemother.* 60 161–167. 10.1128/AAC.01540-15 26482299 PMC4704244

[B19] Lee D. Seo Y. Khan M. S. Hwang J. Jo Y. Son J. (2018). Use of nanoscale materials for the effective prevention and extermination of bacterial biofilms. *Biotech. Bioprocess Eng.* 23 1–10. 10.1007/s12257-017-0348-0

[B20] Lee J.-H. Park J. H. Cho M. H. Lee J. (2012). Flavone reduces the production of virulence factors, staphyloxanthin and α-hemolysin, in *Staphylococcus aureus*. *Curr. Microbiol.* 65 726–732. 10.1007/s00284-012-0229-x 22965624

[B21] Lee J.-H. Regmi S. C. Kim J. A. Cho M. H. Yun H. Lee C. S. (2011). Apple flavonoid phloretin inhibits *Escherichia coli* O157:H7 biofilm formation and ameliorates colon inflammation in rats. *Infect. Immun.* 79 4819–4827. 10.1128/IAI.05580-11 21930760 PMC3232668

[B22] Lee K. Lee J.-H. Ryu S. Y. Cho M. H. Lee J. (2014). Stilbenes reduce *Staphylococcus aureus* hemolysis, biofilm formation, and virulence. *Foodborne Pathog. Dis.* 11 710–717. 10.1089/fpd.2014.1758 25007234

[B23] Liou M. L. Soo P. C. Ling S. R. Kuo H. Y. Tang C. Y. Chang K. C. (2014). The sensor kinase BfmS mediates virulence in *Acinetobacter baumannii*. *J. Microbiol. Immunol. Infect.* 47 275–281. 10.1016/j.jmii.2012.12.004 23453128

[B24] Liu H. Wu Y. Q. Chen L. P. Gao X. Huang H. N. Qiu F. L. (2016). Biofilm-related genes: analyses in multi-antibiotic resistant *Acinetobacter baumannii* isolates from Mainland China. *Med. Sci. Monit.* 22 1801–1807. 10.12659/MSM.898959 27234982 PMC4913728

[B25] Luo L. M. Wu L. J. Xiao Y. L. Zhao D. Chen Z. X. Kang M. (2015). Enhancing pili assembly and biofilm formation in *Acinetobacter baumannii* ATCC19606 using non-native acyl-homoserine lactones. *BMC Microbiol.* 15:62. 10.1186/s12866-015-0397-5 25888221 PMC4381447

[B26] Manoharan R. K. Lee J.-H. Kim Y.-G. Lee J. (2017). Alizarin and chrysazin inhibit biofilm and hyphal formation by *Candida albicans*. *Front. Cell Infect. Microbiol.* 7:447. 10.3389/fcimb.2017.00447 29085811 PMC5650607

[B27] Marti S. Rodriguez-Bano J. Catel-Ferreira M. Jouenne T. Vila J. Seifert H. (2011). Biofilm formation at the solid-liquid and air-liquid interfaces by *Acinetobacter* species. *BMC Res. Notes* 4:5. 10.1186/1756-0500-4-5 21223561 PMC3023692

[B28] Merken H. M. Beecher G. R. (2000). Liquid chromatographic method for the separation and quantification of prominent flavonoid aglycones. *J. Chromatogr. A* 897 177–184. 10.1016/S0021-9673(00)00826-8 11128201

[B29] Mussi M. A. Gaddy J. A. Cabruja M. Arivett B. A. Viale A. M. Rasia R. (2010). The opportunistic human pathogen *Acinetobacter baumannii* senses and responds to light. *J. Bacteriol.* 192 6336–6345. 10.1128/JB.00917-10 20889755 PMC3008525

[B30] National Committee for Clinical Laboratory Standards (2002). *Performance Standards for Antimicrobial Disk and Dilution Susceptibility Tests for Bacteria Isolates From Animals. Approved Standard M31-A2*. Wayne, PA: National Committee for Clinical Laboratory Standards.

[B31] Nelson K. M. Dahlin J. L. Bisson J. Graham J. Pauli G. F. Walters M. A. (2017). The essential medicinal chemistry of curcumin. *J. Med. Chem.* 60 1620–1637. 10.1021/acs.jmedchem.6b00975 28074653 PMC5346970

[B32] Pakharukova N. Tuittila M. Paavilainen S. Malmi H. Parilova O. Teneberg S. (2018). Structural basis for *Acinetobacter baumannii* biofilm formation. *Proc. Natl. Acad. Sci. U.S.A.* 115 5558–5563. 10.1073/pnas.1800961115 29735695 PMC6003481

[B33] Paling F. P. Wolkewitz M. Bode L. G. M. Klein Klouwenberg P. M. C. Ong D. S. Y. Depuydt P. (2017a). *Staphylococcus aureus* colonization at ICU admission as a risk factor for developing *S. aureus* ICU pneumonia. *Clin. Microbiol. Infect.* 23 49.e9–49.e14. 10.1016/j.cmi.2016.09.022 27693658

[B34] Paling F. P. Wolkewitz M. Depuydt P. De Bus L. Sifakis F. Bonten M. J. M. (2017b). *P. aeruginosa* colonization at ICU admission as a risk factor for developing *P. aeruginosa* ICU pneumonia. *Antimicrob. Resist. Infect. Control* 6:38. 10.1186/s13756-017-0197-9 28428877 PMC5397688

[B35] Panche A. N. Diwan A. D. Chandra S. R. (2016). Flavonoids: an overview. *J. Nutr. Sci.* 5:e47. 10.1017/jns.2016.41 28620474 PMC5465813

[B36] Pour N. K. Dusane D. H. Dhakephalkar P. K. Zamin F. R. Zinjarde S. S. Chopade B. A. (2011). Biofilm formation by *Acinetobacter baumannii* strains isolated from urinary tract infection and urinary catheters. *FEMS Immunol. Med. Microbiol.* 62 328–338. 10.1111/j.1574-695X.2011.00818.x 21569125

[B37] Qi L. Li H. Zhang C. Liang B. Li J. Wang L. (2016). Relationship between antibiotic resistance, biofilm formation, and biofilm-specific resistance in *Acinetobacter baumannii*. *Front. Microbiol.* 7:483. 10.3389/fmicb.2016.00483 27148178 PMC4828443

[B38] Rajasekharan S. K. Ramesh S. Satish A. S. Lee J. (2017). Antibiofilm and anti-β-lactamase activities of Burdock root extract and chlorogenic acid against *Klebsiella pneumoniae*. *J. Microbiol. Biotechnol.* 27 542–551. 10.4014/jmb.1609.09043 27974734

[B39] Rajsekharan S. K. Raorane C. J. Lee J. (2018). LED based real-time survival bioassays for nematode research. *Sci. Rep.* 8:11531. 10.1038/s41598-018-30016-5 30069029 PMC6070477

[B40] Runci F. Bonchi C. Frangipani E. Visaggio D. Visca P. (2017). *Acinetobacter baumannii* biofilm formation in human serum and disruption by gallium. *Antimicrob. Agents Chemother.* 61:e01563-16. 10.1128/AAC.01563-16 27799219 PMC5192145

[B41] Russo T. A. Manohar A. Beanan J. M. Olson R. Macdonald U. Graham J. (2016). The response regulator BfmR is a potential drug target for *Acinetobacter baumannii*. *mSphere* 1 e82–e16. 10.1128/mSphere.00082-16 27303741 PMC4888885

[B42] Seifert H. Schulze A. Baginski R. Pulverer G. (1994). Plasmid DNA fingerprinting of *Acinetobacter* species other than *Acinetobacter baumannii*. *J. Clin. Microbiol.* 32 82–86. 8126208 10.1128/jcm.32.1.82-86.1994 PMC262974

[B43] Singh A. K. Prakash P. Singh R. Nandy N. Firdaus Z. Bansal M. (2017). Curcumin quantum dots mediated degradation of bacterial biofilms. *Front. Microbiol.* 8:1517. 10.3389/fmicb.2017.01517 28848526 PMC5552728

[B44] Singh R. (2014). Determination of minimum inhibitory concentration of cycloserine in multidrug- resistant *Mycobacterium tuberculosis* isolates. *Jordan J. Bio. Sci.* 7 139–145. 10.12816/0008228

[B45] Tan Y. Leonhard M. Moser D. Ma S. Schneider-Stickler B. (2018). Antibiofilm efficacy of curcumin in combination with 2-aminobenzimidazole against single- and mixed-species biofilms of *Candida albicans* and *Staphylococcus aureus*. *Colloids Surf. B Biointer.* 174 28–34. 10.1016/j.colsurfb.2018.10.079 30412864

[B46] Teow S. Y. Liew K. Ali S. A. Khoo A. S. B. Peh S. C. (2016). Antibacterial action of curcumin against *Staphylococcus aureus*: a brief review. *J. Trop. Med.* 2016:2853045. 10.1155/2016/2853045 27956904 PMC5124450

[B47] Thompson R. J. Bobay B. G. Stowe S. D. Olson A. L. Peng L. Su Z. (2012). Identification of BfmR, a response regulator involved in biofilm development, as a target for a 2-aminoimidazole-based antibiofilm agent. *Biochemistry* 51 9776–9778. 10.1021/bi3015289 23186243 PMC3567222

[B48] Tomaras A. P. Dorsey C. W. Edelmann R. E. Actis L. A. (2003). Attachment to and biofilm formation on abiotic surfaces by *Acinetobacter baumannii*: involvement of a novel chaperone-usher pili assembly system. *Microbiology* 149 3473–3484. 10.1099/mic.0.26541-0 14663080

[B49] Tyagi P. Singh M. Kumari H. Kumari A. Mukhopadhyay K. (2015). Bactericidal activity of curcumin I is associated with damaging of bacterial membrane. *PLoS One* 10:e0121313. 10.1371/journal.pone.0121313 25811596 PMC4374920

[B50] Uppuluri P. Lin L. Alqarihi A. Luo G. Youssef E. G. Alkhazraji S. (2018). The Hyr1 protein from the fungus *Candida albicans* is a cross kingdom immunotherapeutic target for *Acinetobacter* bacterial infection. *PLoS Pathog.* 14:e1007056. 10.1371/journal.ppat.1007056 29746596 PMC5963808

[B51] Wu H. Moser C. Wang H. Z. Hoiby N. Song Z. J. (2015). Strategies for combating bacterial biofilm infections. *Int. J. Oral Sci.* 7 1–7. 10.1038/ijos.2014.65 25504208 PMC4817533

