# Supplementary material for: Antibiofilm and Antivirulence Efficacies of Flavonoids and Curcumin Against Acinetobacter baumannii
Source: Front Microbiol. 2019 May 8;10:990. doi: 10.3389/fmicb.2019.00990 (PMC6517519; doi:10.3389/fmicb.2019.00990)
Supplement: Supplementary file 1 [file Data_Sheet_1.docx]

**Supplementary materials**

**Antibiofilm and antivirulence efficacies of flavonoids and curcumin against *Acinetobacter baumannii***

**Authors:**

Chaitany Jayprakash Raorane^a,#^, Jin-Hyung Lee^a,#^, Yong-Guy Kim^a^, Satish Kumar Rajasekharan^a^, Rodolfo García-Contreras^b^, and Jintae Lee^a,^*

***Corresponding author:** E-mail: [jtlee@ynu.ac.kr](mailto:jtlee@ynu.ac.kr)

**Supplementary Figure S1. Effects of curcumin on motility and pellicle formation of *A. baumannii*** **clinical isolates.** The surface motility on 0.3% agar of *A. baumannii* clinical isolates, A 550 (A), A 556 (B), and A 580 (C) was investigated after adding curcumin at 10 or 50 µg/ml. The pellicle formation of clinical isolates was performed in the presence or absence of curcumin (50 µg/ml) for 72 h at 25 °C (D). The bar graphs represent surface motility diameters in cm in the presence and absence of curcumin (E). Pellicle formation as determined by spectrophotometry at OD_600_ (F). Experiments were performed using at least two independent cultures. **p <* 0.05 versus untreated controls.

**
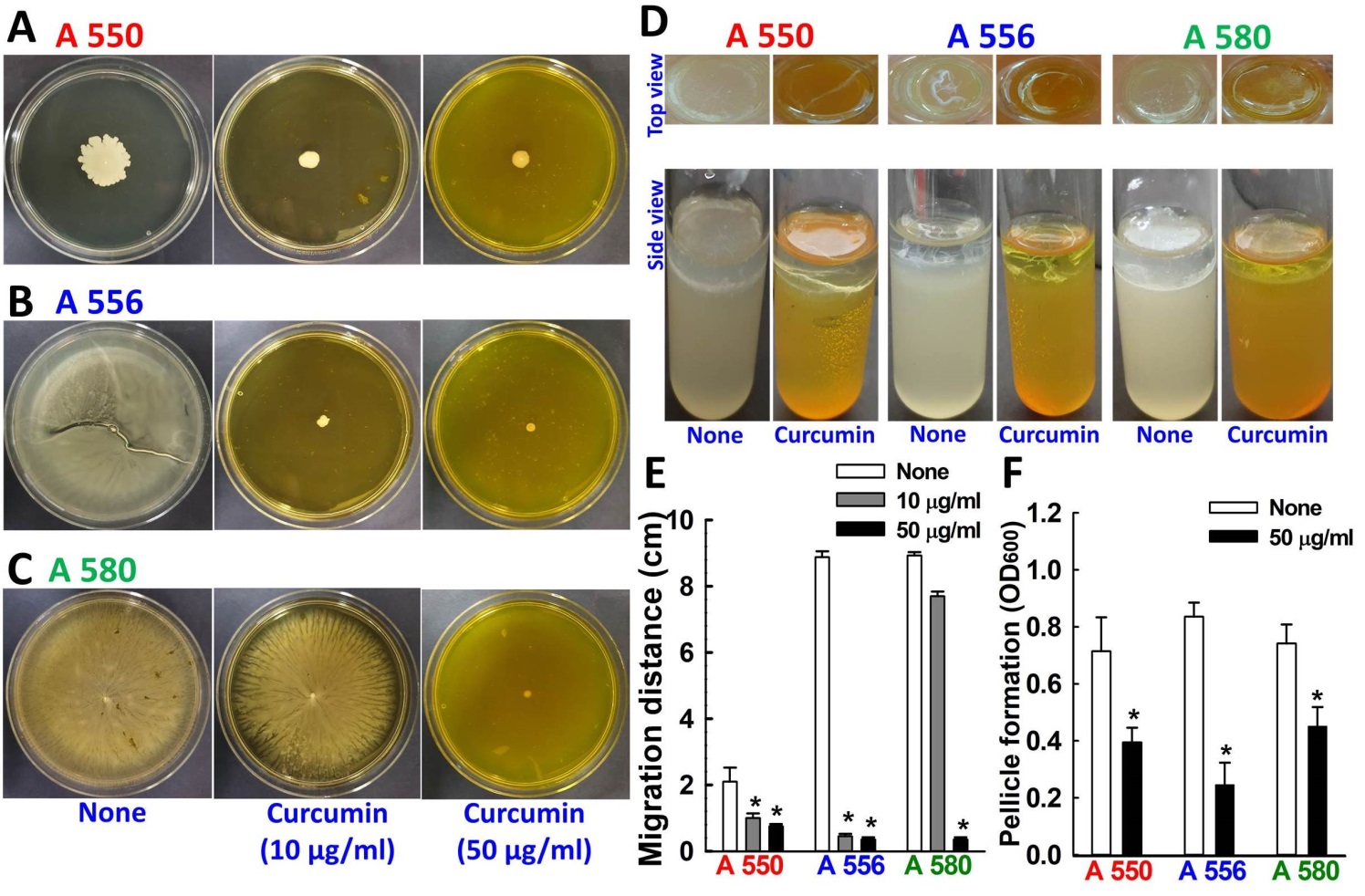
**

**Supplementary Figure S2.** 2D Interaction patterns of flavonoids with the N-terminal domain of BfmR. Yellow dotted lines in 2D interaction diagrams denote hydrogen bonds. Negatively charged amino acids are depicted as red drops, hydrophobic amino acids as light green drops, and positively charged amino acids as violet drops. Pi-Pi stacking is shown as red lines.


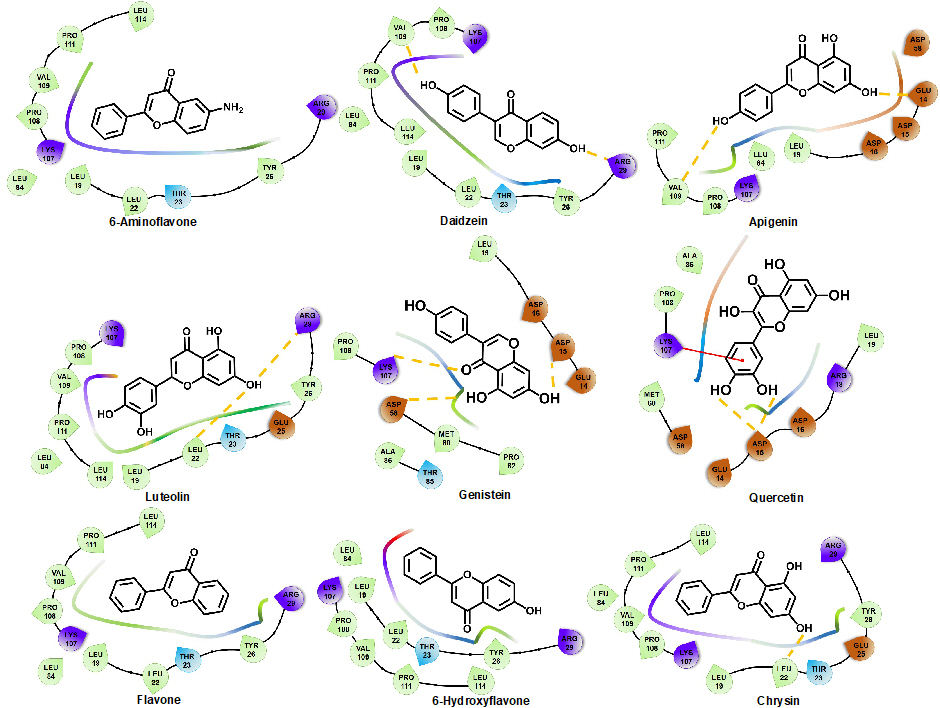


**Supplementary Figure S3.** **Effect of curcumin on the survival of nematodes with *A*. *baumannii* and/or *C. albicans* infections.** Light microscopic images of adult *C. elegans*, showing live nematodes in none and curcumin treated groups, and dead worms in *A*. *baumannii, C. albicans,* or mixed culture infected groups.


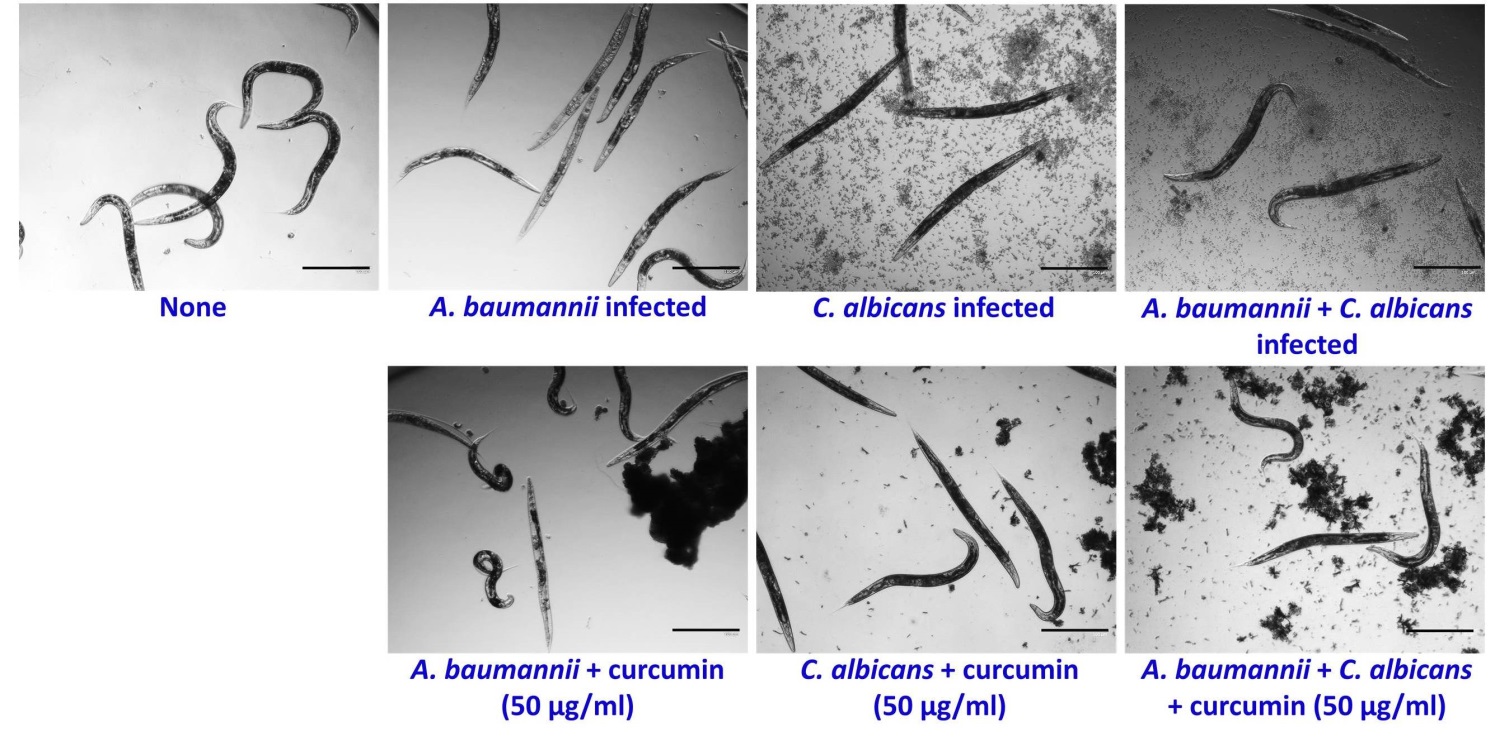


**Supplementary Figure S4. Post CFU value of *A*. *baumannii* in the *C. elegans* infection model.** CFU was measured at 0, 4 and 8 days of post infection. Four independent cultures were used.


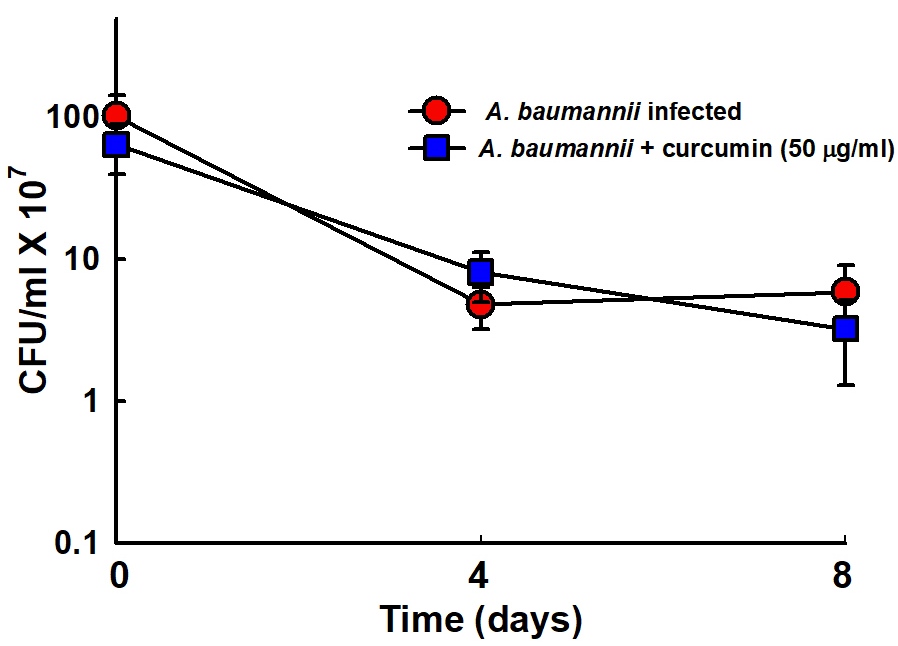


**Supplementary Table S1. Molecular docking parameters and its correlation with biofilm formation. N/D = not determined.**

| Chemical | No. of H-bond | Key residue | Pi-Pi stacking | % of biofilm formation (50 µg/ml) | Binding energy score (kcal/mol) |
| --- | --- | --- | --- | --- | --- |
| 2-Aminoimidazole | 2 | Asp15, Asp58 | Lys107 | N/D | -25.7 |
| LED209 | 1 | Asp16 | Lys107 | N/D | -44.1 |
| Virstatin | 2 | Asp16, Lys107 | - | N/D | -37.4 |
| None | - | - | - | 100 ± 5 | - |
| 6-Aminoflavone | - | - | - | 150 ± 4 | -30.2 |
| Apigenin | 2 | Glu14, Val109 |  | 133 ± 6 | -32.1 |
| 6-Hydroxyflavone | - | - | - | 126 ± 3 | -37.3 |
| Chrysin | 1 | Leu22 | - | 120 ± 2 | -37.9 |
| Flavone | - | - | - | 102 ± 3 | -29.2 |
| Daidzein | 2 | Arg29, Val109 | - | 92 ± 6 | -35.4 |
| Luteolin | 2 | Leu22, Arg29 | - | 68 ± 5 | -39.7 |
| Genistein | 3 | Asp15, Asp58, Lys107 | - | 48 ± 6 | -45.2 |
| Quercetin | 2 | Asp15 (2) | Lys107 | 47 ± 8 | -38.6 |
| Fisetin | 2 | Asp15 (2) | Lys107 | 29 ± 1 | -35.4 |
| Phloretin | 2 | Asp15, Asp58 | Lys107 | 21 ± 10 | -41.8 |
| Curcumin | 3 | Asp15, Val109 (2) | - | 23 ± 8 | -38.7 |
